# Development of nanobody-horseradish peroxidase-based sandwich ELISA to detect *Salmonella Enteritidis* in milk and in vivo colonization in chicken

**DOI:** 10.1186/s12951-022-01376-y

**Published:** 2022-03-31

**Authors:** Kui Gu, Zengxu Song, Changyu Zhou, Peng Ma, Chao Li, Qizhong Lu, Ziwei Liao, Zheren Huang, Yizhi Tang, Hao Li, Yu Zhao, Wenjun Yan, Changwei Lei, Hongning Wang

**Affiliations:** 1 Key Laboratory of Bio-Resource and Eco-Environment of Ministry of Education, College of Life Sciences, Sichuan University, Chengdu, Sichuan People’s Republic of China; 2 Animal Disease Prevention and Food Safety Key Laboratory of Sichuan Province, Chengdu, People’s Republic of China; 3 State Key Laboratory of Biotherapy and Cancer Center, West China Hospital, Sichuan University, Chengdu, 610041 China

**Keywords:** *S. Enteritidis*, Nanobody, Nanobody-horseradish peroxidase, Phage display technology, Immunoassay

## Abstract

**Background:**

*Salmonella Enteritidis* (*S. Enteritidis*) being one of the most prevalent foodborne pathogens worldwide poses a serious threat to public safety. Prevention of zoonotic infectious disease and controlling the risk of transmission of *S. Enteriditidis* critically requires the evolution of rapid and sensitive detection methods. The detection methods based on nucleic acid and conventional antibodies are fraught with limitations. Many of these limitations of the conventional antibodies can be circumvented using natural nanobodies which are endowed with characteristics, such as high affinity, thermal stability, easy production, especially higher diversity. This study aimed to select the special nanobodies against *S. Enteriditidis* for developing an improved nanobody-horseradish peroxidase-based sandwich ELISA to detect *S. Enteritidis* in the practical sample. The nanobody-horseradish peroxidase fusions can help in eliminating the use of secondary antibodies labeled with horseradish peroxidase, which can reduce the time of the experiment. Moreover, the novel sandwich ELISA developed in this study can be used to detect *S. Enteriditidis* specifically and rapidly with improved sensitivity.

**Results:**

This study screened four nanobodies from an immunized nanobody library, after four rounds of screening, using the phage display technology. Subsequently, the screened nanobodies were successfully expressed with the prokaryotic and eukaryotic expression systems, respectively. A sandwich ELISA employing the SE-Nb9 and horseradish peroxidase-Nb1 pair to capture and to detect *S. Enteritidis*, respectively, was developed and found to possess a detection limit of 5 × 10^4^ colony forming units (CFU)/mL. In the established immunoassay, the 8 h-enrichment enabled the detection of up to approximately 10 CFU/mL of *S. Enteriditidis* in milk samples. Furthermore, we investigated the colonization distribution of *S. Enteriditidis* in infected chicken using the established assay, showing that the *S. Enteriditidis* could subsist in almost all parts of the intestinal tract. These results were in agreement with the results obtained from the real-time PCR and plate culture. The liver was specifically identified to be colonized with quite a several *S. Enteriditidis*, indicating the risk of *S. Enteriditidis* infection outside of intestinal tract.

**Conclusions:**

This newly developed a sandwich ELISA that used the SE-Nb9 as capture antibody and horseradish peroxidase-Nb1 to detect *S. Enteriditidis* in the spike milk sample and to analyze the colonization distribution of *S. Enteriditidis* in the infected chicken. These results demonstrated that the developed assay is to be applicable for detecting *S. Enteriditidis* in the spiked milk in the rapid, specific, and sensitive way. Meanwhile, the developed assay can analyze the colonization distribution of *S. Enteriditidis* in the challenged chicken to indicate it as a promising tool for monitoring *S. Enteriditidis* in poultry products. Importantly, the SE-Nb1-vHRP as detection antibody can directly bind *S. Enteritidis* captured by SE-Nb9, reducing the use of commercial secondary antibodies and shortening the detection time. In short, the developed sandwich ELISA ushers great prospects for monitoring *S. Enteritidis* in food safety control and further commercial production.

**Graphic Abstract:**

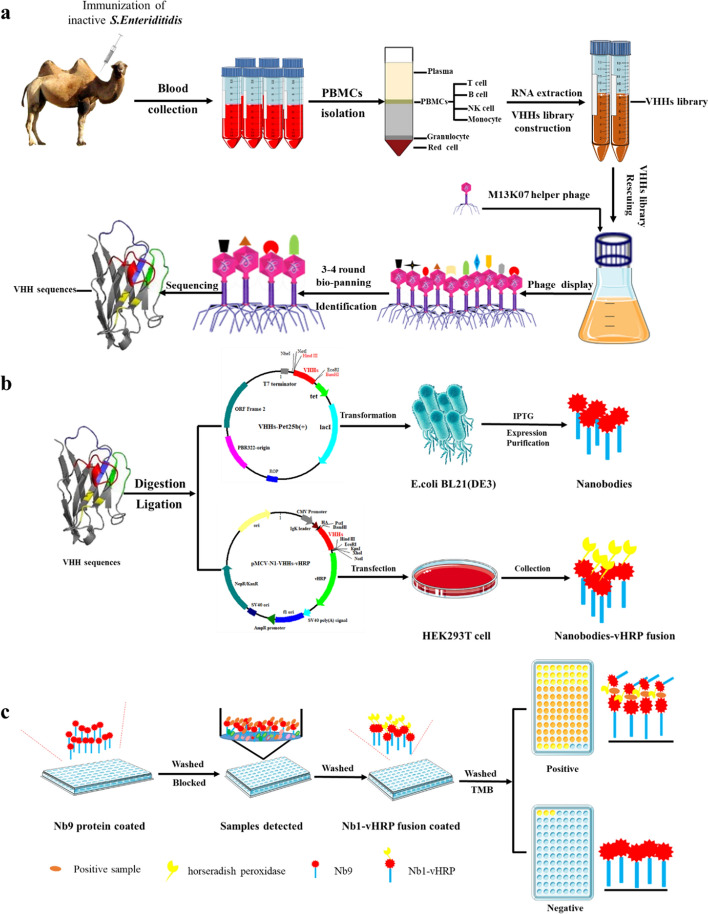

**Supplementary Information:**

The online version contains supplementary material available at 10.1186/s12951-022-01376-y.

## Background

Nanobodies (Nbs) are single-domain antibodies (sdAbs) derivatives of the immunoglobulin heavy chain in camelids or sharks, which represent the smallest antibody unit with complete antigen-binding characteristics owing to about 15 kDa molecular mass [1]. Compared to the conventional antibodies, the natural nanobodies are endowed with special characteristics, like high affinity, thermal stability, easy production, higher diversity and improved ability to detect cryptic epitope due to the longer complementary determining region 3 (CDR3) [2–4]. Several recent studies have demonstrated the small size nanobodies to be widely used in detecting the target antigen, like the virus [5–8], bacteria [9], parasite [10], toxins [11–13] and others [14, 15].

Among all the foodborne pathogens, *Salmonella Typhimurium (S. Typhimurium)* and *Salmonella Enteritidis (S. Enteritidis)* are well-known to be the most harmful zoonotic pathogens [16]. Studies have shown that *S. Enteritidis* initiate significant cases comprising 1.35 million infections, 26,500 hospitalizations, and 420 deaths annually in America [17]. Hence, food safety regulations in many countries demand that strict surveillance of *S. Enteritidis* should compulsorily be undertaken in foodborne products [17, 18]. As a result, rapid and sensitive detection technology should be devised for monitoring *S. Enteritidis*. Such an advanced detection technology would provide a significant measure for reducing the prevalence of *S. Enteritidis* and the transmission risk to humans, as well as prevent and avoid the foodborne infectious diseases [19]. Nanobodies have robust properties, which make immunoassays based on special nanobodies a feasible and promising option for monitoring *S. Enteritidis*.

There are many reported studies where nanobodies-fused reporter proteins have been used as detection agents for detecting target antigen by developing immunoassays to develop the immunoassays like the competitive ELISA and sandwich ELISA [20–22]. The reporter-nanobody fusions have been reported to exhibit a high affinity and have been employed in assays without requiring a second antibody, which would effectively shorten the time and lower the use of the reagent [1, 23].

**Scheme 1:**
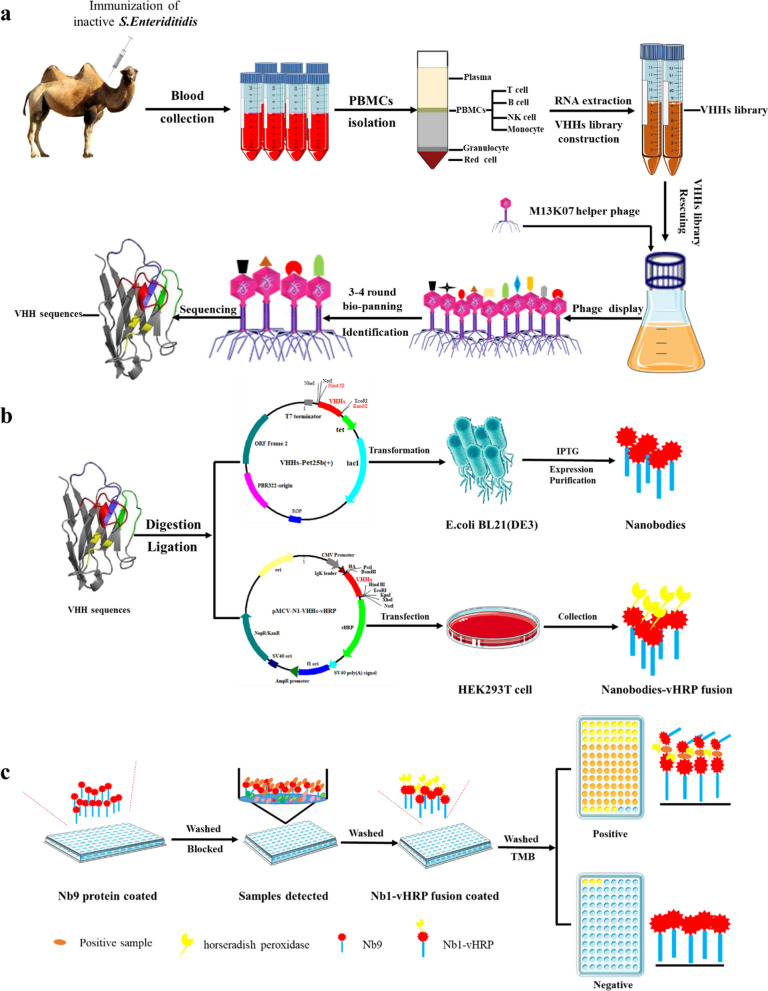
Graphic abstract of the developed sandwich to detect *S. Enteritidis* in practical sample. **a** Nbs were screened by the phage display platform. **b** SE-Nbs and SE-Nbs-vHRP were produced by *E. coli* expression system and eukaryotic expression system in HEK293T cell, respectively. **c** Detection practical sample with the developed sandwich ELISA

The merit of nanobodies was harnessed for detecting *S. Enteritidis* by developing an improved nanobody-horseradish peroxidase-based sandwich ELISA. The phage display technology was used to screen the specific nanobodies against *S. Enteritidis* obtained from an immunized Bactrian camel (Scheme 1a). Based on the coding sequence of nanobodies, the His-tagged Nbs and nanobodies-horseradish peroxidase were produced using prokaryotic and eukaryotic expression systems, respectively (Scheme 1b). In this newly developed sandwich ELISA, a His-tagged Nb was used as the capturing antibody while the nanobodies-horseradish peroxidase was used as detecting antibodies to detecting *S. Enteritidis* in the practical sample, such as the spiked milk samples (Scheme 1c). Furthermore, the developed immunoassay was used to evaluate the colonization of *S. Enteritidis* in the intestinal tract and organs of chickens, showing high agreement with the real-time PCR. Moreover, this established assay was found to not require the use of a secondary antibody, time and cost saving and exhibited a promising prospect for monitoring *S. Enteritidis* in controlling food safety.

## Materials and methods

### Materials and reagents

The double blood bags used blood collection were obtained from Suzhou Laishi Transfusion Equipment Co., Ltd (Suzhou, China). The complete Freund’s adjuvant, incomplete Freund’s adjuvant and Amicon UltraCentrifugal Filter Units were procured from Sigma Aldrich (St. Louis, MO, USA). All the restriction enzymes utilized in the study were procured from New England Biolabs (Beijing) LTD (Beijing, China). The 96-well microplates were purchased from Corning (New York, NY, USA). The PCR Purification Kit, Gel Extraction Kit and TIANprep Mini Plasmid Kit used for plasmid preparation were obtained from TIANGEN (Beijing, China). All the reagents utilized in this study were analytical grade, unless otherwise indicated.

### Cells, strains and vectors

The HEK293T cell lines were cultured in the Dulbecco’s modified Eagle’s medium (Life Technologies Corp, USA) supplemented with 10% fetal bovine serum (FBS, Gibco USA) and 100 IU/mL penicillin‒streptomycin solution (Gibco USA). All the strains were from our laboratory preserved strains (Animal Disease Prevention and Food Safety Key Laboratory of Sichuan Province, Chengdu, China) including *S. Enteriditidis* FY-04 (GenBank accession JAKIRO000000000), *S. Enteriditidis* ATCC13076, *S. Typhimurium* ATCC23566, *S. Pullorum* ATCC13036, *S. gallinarum* CICC21510, *Staphylococcus aureus* ATCC25923, *Klebsiella pneumoniae* (isolated by Laboratory), *Campylobacter jejuni* (isolated by Laboratory), *Listeria monocytogenes* ATCC19115, *Escherichia coli* ATCC 25922. The pET-25b vector (Novagen, USA) was utilized to express the recombinant nanobody fusions against *S. Enteriditidis* in the *E. coli* system. The pMECS vector and M13K07 helper phage were kindly gifted by Ph.D. Qizhong Lu from the Stata Key Laboratory of biotherapy, Sichuan University, and were used to construct the VHH library. The pEGFP-N1 vector (Clontech, Japan) stored at in our laboratory was used as a backbone to construct the pCMV-N1-HRP vector.

### Inactivation of the *S. Enteriditidis* strain

To ensure the safety of the immunized Bactrian camel, the pathogenic *S. Enteritidis* FY-04 was inactivated with formaldehyde as previously described [24]. Briefly, the *S. Enteriditidis* was cultured overnight in LB media and harvested by centrifugation at 8000 rpm for 10 min and resuspended in PBS supplemented with 0.2% (v/v) formaldehyde, and placed at 37℃ for 48 h. The inactivated *S. Enteriditidis* was adjusted to 1 × 10^9^ CFU/mL of the final concentration. Finally, the inactivation efficacy was evaluated using the plating count. There were no colonies on the plate, suggesting that the strains were completely inactivated and could be used to inject the Bactrian camel for the experiment.

### Bactrian camel immunization and VHH library construction

To obtain a specific VHH library against *S. Enteriditidis* FY-04, a previously described protocol was used [16, 25]. Briefly, a healthy 4–year-old Bactrian camel was subcutaneously immunized five times with the inactivated *S. Enteritidis* FY-04 mixed with equal volumes of adjuvant. Freund’s complete adjuvant was used for the first immunization, four times followed with Freund’s incomplete adjuvant at an interval of 2 weeks. To evaluate the titration of serum from the last immunized camel, an indirect ELISA with HRP-conjugated rabbit anti-camel IgG (SE283, Solarbio, Beijing, China) was performed where the inactivated *S. Enteritidis* as a coated antigen having a concentration of 1 × 10^9^ CFU/mL was used.

**Scheme 2:**
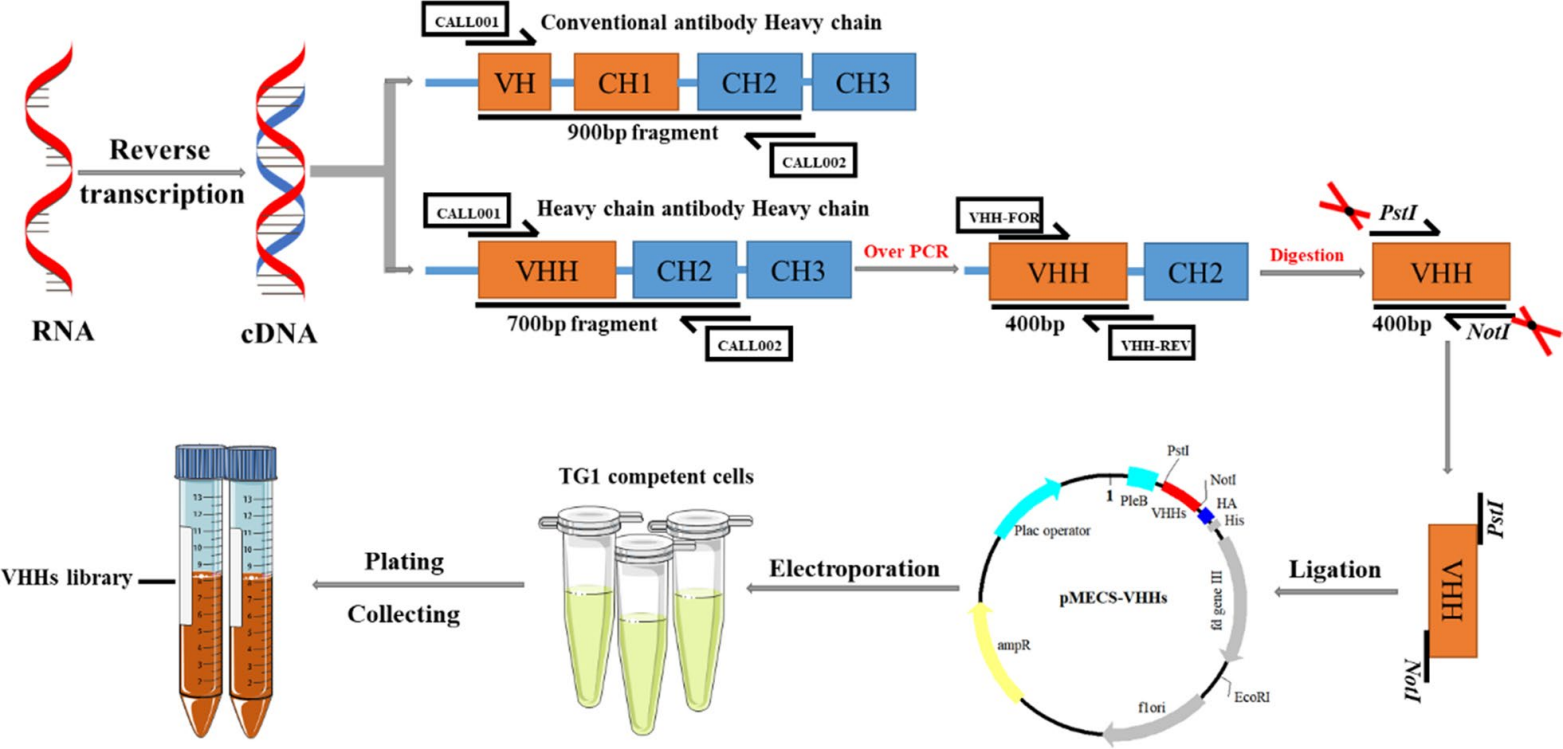
Construction of phage-displayed library including two rounds PCR, digestion, ligation, electroporation, plating and collecting

Three-four days after the last immunization, the peripheral blood mononuclear cell was collected from a 200 mL blood sample. The cDNA prepared from the total RNA was used for constructing the nanobodies (VHH) library using the procedures presented in Scheme 2. Briefly, the VHH fragments were amplified by a two-steps PCR, with primer pairs listed in Additional file 1: Table S1. The CALL001 and CALL002 were the first-round PCR amplification pair of primers, while VHH-FOR and VHH-REV constituted the second pair of primers [26]. The fragments of the second PCR were ligated into the phagemid vector pMECS (digested by the restriction enzyme *PstI* and *NotI*). Then, the recombinant phagemids (pMECS-VHHs) were electroporated into the pre-prepared *E. coli strain TG1* electroporation-competent cells. The capacity of the VHH library constructed here was analyzed through the plate count method, and the LB plates were supplemented with 2% (w/v) final concentration of glucose and 100 µg/mL ampicillin. The randomly-selected 48 colonies were determined using the PCR amplification with the primer pair MP57 and GIII (Additional file 1: Table S1). Subsequently, the positive colonies were sequenced for identifying diversity. Finally, the VHH library was stored at −80 ℃ in the LB medium supplemented with 20% glycerol and 100 µg/mL ampicillin until further use.

### Screening and identification of the specific nanobodies against *S. Enteriditidis*

The specific nanobodies were selected against *S. Enteriditidis* across four rounds of bio-panning using indirect ELISA (iELISA) as described previously [24, 27]. Briefly, a representative aliquot of the VHH library was cultured and infected with the M13K07 helper phages to obtain the rescue phage in every round of selection. The microtiter plates were coated with the inactivated*-S. Enteriditidis* of 1 × 10^8^ CFU/well in NaHCO_3_ buffer (100 mM, pH = 8.2) at 4℃ overnight. The coated wells were washed three times with PBS containing 2% tween-20 (PBST, v/v), and then blocked with 3% skimmed milk (w/v). Then, 5 × 10^11^ PFU of the rescue phage was added and incubated at 37℃ for 1 h. Then, each well was washed ten times with PBST, 100 μL TEA solution (100 mM triethylamine, pH = 11.0) was added and incubated at RT for 10 min to elute specific phage particles, which was immediately neutralized with 100 μL of 1.0 M Tris–HCl (pH = 7.4). Subsequently, for the next round of selection, the eluted phage particles were transferred to infect the TG1 cells for titration evaluation and amplification. The population of TG1 cells infected was counted to quantify the input and output phages and the enriched phage particles were detected using iELISA with an anti-M13 antibody (Hangzhou HuaAn Biotechnology Co., Ltd, Hangzhou, China). After four rounds of screening, 96 individual clones were randomly selected from the third, fourth round of eluted phages. They were then separately induced with 1 mmol/L IPTG for expressing the soluble nanobodies in the *E. coli* periplasm in the 96-well plates. Several freeze–thaw cycles yielded the periplasmic extract (PE) which consisted of Nbs with Hemagglutinin (HA) and His tags. Furthermore, the presence of specific nanobodies against *S. Enteriditidis* were determined using iELISA with mouse anti-HA monoclonal antibody (Sino Biological, Inc, Beijing, China). Finally, sequencing based on their complementary determining regions (CDRs) amino acid sequence were able to identify and classify positive colonies (P/N > 3.0).

### Expression, purification and characterization of the nanobodies against *S. Enteriditidis*

To obtain nanobodies against *S. Enteriditidis*, the pET-25b^+^ vector with a signal sequence for expressing C-terminally HSV-tagged and His-tagged proteins in the periplasm was utilized for expressing high-yield nanobodies [23, 28]. The VHH genes were amplified by PCR using SE-Nb-F and SE-Nb-R primer pairs (as listed in Additional file 1: Table S1). To develop recombinant plasmids, named the pET-25b^+^-VHHs vector, the PCR products were digested and ligated into the pET-25b^+^ vector at the same restriction site. The *E. coli* BL21 (DE3) transformed positive vectors were induced with 0.2 mM IPTG for 16 h at 16 °C to obtain the nanobodies against *S. Enteriditidis*. Moreover, to evaluate the binding capacity of periplasm extract, an iELISA with anti-HSV tag monoclonal antibody (Bioss Inc, Beijing, China) was used. The Nbs were purified from periplasm extract using Ni-IDA 6FF Sefinose (TM) Resin Kit and imidazole were removed using the Sephadex DeSalting Gravity Column (Sangon Biotech, Shanghai, China). The expression and purification of the nanobodies was analyzed using SDS-PAGE and western blot. The binding capacity, specificity and cross-reactivity of the purified nanobodies, for the strains which include one *S. Enteriditidis*, and the other three *Salmonellas*, and five *non-Salmonellas* were confirmed by iELISA using the anti-HSV tag monoclonal antibody as a detection antibody.

### Construction of the pCMV-N1-vHRP vector and producing nanobody‑HRP fusions against *S. Enteriditidis*

#### Construction and characterization of the pCMV-N1-vHRP vector

The pCMV-N1-vHRP vector was developed using the pEGFP-N1 vector as a backbone. This vector is designed to express nanobody fusions including the human Ig kappa chain signal peptide, HA tag, nanobodies coupled with the codon-optimized HRP, and the His tag in the HEK293T cells [20, 21, 23]. Briefly, the components like the secreting signal sequence (the human IgG kappa chain), HA tag, multiple cloning site (MCS), a short linker, vHRP coding sequence and 7 × His tag were synthesized by Sangon Biotech. Meanwhile, the validated sequence was completely digested using the enzymes *NheI* and *XbaI* before it was ligated into the commercial vector pEGFP-N1 (cut with the same enzymes) to create the pCMV-N1-vHRP vector. To determine whether inserting an exogenous gene could successfully express upon recombination pCMV-N1-vHRP vector in HEK293T cells, the EGFP coding sequence was amplified as a positive control using the primer pairs EGFP-F and EGFP-R (Additional file 1: Table S1) and subsequently inserted into the pCMV-N1-vHRP vector using the restriction enzymes *PstI* and *HindIII*. The recombination vector named pCMV-EGFP-vHRP was transfected into the HEK293T cell using Lipo8000™ Transfection Reagent (Beyotime Biotechnology, Shanghai, China). After 48 h of transfection, the EGFP-vHRP fusion was directly observed using fluorescence microscopy (Leica DMi8, Germany).

#### Expression and characterization of the nanobody‑vHRP fusion against *S. Enteriditidis*

The nanobody-HRP fusions were expressed in the HEK293T cells as described above. Briefly, the VHH coding genes were amplified by PCR using the Nb‑vHRP-F and Nb‑vHRP-R primers (Additional file 1: Table S1) and then ligated into the pCMV-N1-vHRP vector, to be ultimately named as pCMV-Nbs-vHRP. The positive plasmids were transfected to the HEK293T cells, and after 72 h of transfection, the supernatant of the culture was collected by centrifuging at 1000×*g* for 5 min to remove the cell debris. The secreted nanobody-HRP fusions supplemented with 0.02% NaN_3_ (w/v) were then stored at 4℃ for direct use. Then, the expressions of the nanobody-HRP fusions in the HEK293T cells and culture supernatant were separately determined using indirect immunofluorescence assay (IFA) and Western blot assay. In addition, the anti-HA monoclonal antibody was not only treated as the first antibody with the FITC- and HRP-labeled goat anti-mouse antibodies (PROTEINTECH GROUP, Wuhan, China) but also as the second antibody in the two assays above. To evaluate the specificity, titers and cross-reactivity of the nanobody-HRP fusions in the cultured supernatant, the iELISA was performed using the anti-HA monoclonal antibody as the second antibody.

### Indirect ELISA

The iELISA being a common immunoenzyme technique was frequently used in this studying for detecting the titer in the camel blood samples, screening of the specific nanobodies, and the specific binding and cross- reactivity of the nanobodies and nanobody-HRP fusions. Briefly, the inactivated *S. Enteritidis* or other strains (1 × 10^8^ CFU/well) were coated as antigens in the 96-well plate overnight at 4 ℃, where the NaHCO_3_ buffer was used as a negative control. After blocking with 3% skimmed-milk, the primary antibodies (sera of different dilutions, the nanobodies of prokaryotic expression, periplasmic extracts, and the nanobody-HRP fusions) were added to the plates and incubated for 1 h at 37 ℃. For directly detecting the titer of the serum samples, the HRP-conjugated rabbit anti-camel antibody was employed as a detection antibody for direct use. The anti-HA and anti-HSV monoclonal antibodies were separately treated as the second antibody in the specific nanobodies screening and characterization, which were both followed by treatment with the HRP-conjugated goat anti-mouse IgG at 37℃ for 1 h. However, in the case of the nanobody-HRP fusions, no secondary and detection antibodies were used. After washing five times with PBST, 100 μL/well of TMB (Solarbio, Beijing, China) was added and incubated in the dark at 37 °C for 15 min for a colorimetric reaction, followed by stopping with 2 mol/L H_2_SO_4_ (50 μL/well). Finally, the optical density was measured at 450 nm using a Multiskan FC microplate reader (Thermo Fisher Scientific, USA).

### Immunofluorescence assay (IFA)

The procedure for IFA was modified based on a previously reported assay [23]. After 48 h of transfection, the transfected cells were fixed with 4% paraformaldehyde at RT for 30 min. Then, the cells were blocked with 2% bovine serum albumin and washed three times with PBS. The anti-HA monoclonal and FITC-conjugated goat anti-mouse antibodies were then used as the first and the second antibodies for incubating at 37 °C for 1 h. After DAPI staining, the cells were directly observed under a fluorescence microscope.

#### Establishment of the double nanobody‑based sandwich ELISA

The double nanobody‑based sandwich ELISA was established for detecting *S. Enteriditidis*, where the His-tagged Nbs and nanobody-HRP fusions were used as for capturing and detecting antibodies, respectively. The orthogonal assay was designed to select the best pair, procedure based on a previously reported procedure [24, 29]. Briefly, the plates were coated with the His-tagged Nbs (800 ng/well) overnight at 4 °C. After blocking with 3% (w/v) skim-milk for 1 h, the *S. Enteriditidis* (1 × 10^8^ CFU/well) was added and incubated in the plates for 1 h at 37 °C, an equal volume of NaHCO_3_ buffer as the negative control. To this, 100 μL of different nanobody-HRP fusions (1:10 dilution ratio of original supernatant) were added to the plates and incubated for 1 h at 37 °C. The TMB (100 uL/well) which was used as a substrate of HRP was added to the plates and incubated in dark for 15 min at 37 °C. The color reaction was then stopped by adding 2 mol/L H_2_SO_4_ (50 μL/well) and the OD_450_ nm value of each well was measured with a microplate reader (Thermo Fisher Scientific, USA). Henceforth, the best pair of nanobodies with the highest P/N value was selected. The optimal concentration for the capturing and detecting Nbs was determined for the different concentrations of the coated antigen by searching for the conditions with the highest P/N value as previously described [22]. Briefly, the sandwich ELISA was performed employing different amounts of the His-tagged Nbs (200, 400, 600, 800, 1000, 1500, 2000, 2500 ng/well) and the different dilution ratio of the nanobody-HRP fusions (1:1, 1:5, 1:10, 1:20, 1:50, 1:100, 1:200 and 1:1000). *S. Enteriditidis* was employed as the positive control while NaHCO_3_ buffer served as the negative control. Then, when the P/N ratio was the highest, the optimal amount of the capture nanobody and detection nanobody-HRP fusions were determined. To characterize the specificity and cross-reactivity of the double nanobody‑based sandwich ELISA, one *S. Enteriditidis*, the other three *Salmonella* and five non-*Salmonella* as mentioned above were used to evaluate. The standard curves were determined to quantify and determine the limit of detection (LOD) of the developed method. It used 3 aliquots of gradient dilution of *S. Enteriditidis* as detection antigen, with a primary concentration of 3.0 × 10^9^ CFU/mL.

#### Detection of *S. Enteriditidis* spiked in the milk

To validate the effectiveness of the double nanobody‑based sandwich ELISA, the skimmed milk from local supermarket ensured to be free of *S. Enteriditidis* using the plate counting method. The spiked milk sample was prepared by adding different concentrations of the *S. Enteritidis* FY-04 to the milk to attain final concentrations of 1 × 10^6^, 1 × 10^7^ and 1 × 10^8^ CFU/mL, respectively. The prepared samples were collected by centrifugation at 5000 rpm for 10 min. And after washing the pellet with PBS, the established sandwich ELISA was used to analyze the recovery rate. For detecting the enriched bacteria, 1 mL spiked milk with *S. Enteritidis* around 10 CFU were mixed with 9 mL LB liquid medium and enriched at 37 °C for 6 h, 8 h, 10 h, 12 h, 14 h at 37 °C, respectively. The samples were analyzed using the same method as described above, without adding *S. Enteritidis* to the milk sample as a negative control.

#### Analyzing of *S. Enteritidis* in vivo colonization in Chicken

The neonatal chickens were randomly divided into two groups under feeding conditions where Group A was orally challenged with 1.0 × 10^8^ CFU of *S. Enteritidis* FY-04, while group B was treated with the same volume of PBS as the blank control. On the fourth day after the oral challenge, all the chickens from each group were dissected to collect the tissues like the heart, liver, stomach, duodenum, jejunum, ileum, caecum, colon, rectum, pancreas and kidney. For bacterial enrichment of these selected tissues, 1 g of every sample was cut and mixed with 25 mL pre-prepared buffered peptone water (BPW) and enriched at 37 °C for 12 h. Subsequently, the samples were centrifuged at 5000 rpm for 10 min and then were resuspended in PBS. The samples containing *S. Enteritidis* were determined using the established sandwich ELISA, qPCR detection and plate culture.

#### Real-time PCR

The bacteria genomic DNA was extracted using TIANamp Bacteria DNA Kit (Beijing, China) following the manufacturer's instructions. The real-time PCR reactions were executed according to previous descriptions [30]. Precisely, the reaction mixture comprised 10 μL of SsoFast EvaGreen Supermix, 1 μL (10 μM) of each primer (qPCR-F and qPCR-R as listed in Additional file 1: Table S1), 2 μL of the DNA template, were added along with sterilized water to reach the final reaction volume of 20 μL. Subsequently, the assay was performed at a temperature of 95 °C for 2 min, with 40 cycles of 95 °C for 5 s, followed by 60 °C for 10 s, 72 °C for 20 s, and the fluorescence was assessed at 72 °C at the end of each cycle, calculating the Ct values (the cycle at which the fluorescence exceeds the background level). In this assay, the genomic DNA of *S. Enteritidis* ATCC 13,076 was used as a positive template and sterilized water as a negative control.

#### Statistical analysis

All the assays were independently repeated at least three times. The data presentation was represented using GraphPad Prism version 7.0 (GraphPad Software, San Diego, CA, USA). The Kappa values were calculated to estimate the coincidence between the developed sandwich ELISA assays, and the real-time PCR using the SPSS software (Version 20, http://www.spss.com.cn).

## Results

### Construction of the VHH library against *S. Enteriditidis*

**Fig. 1:**
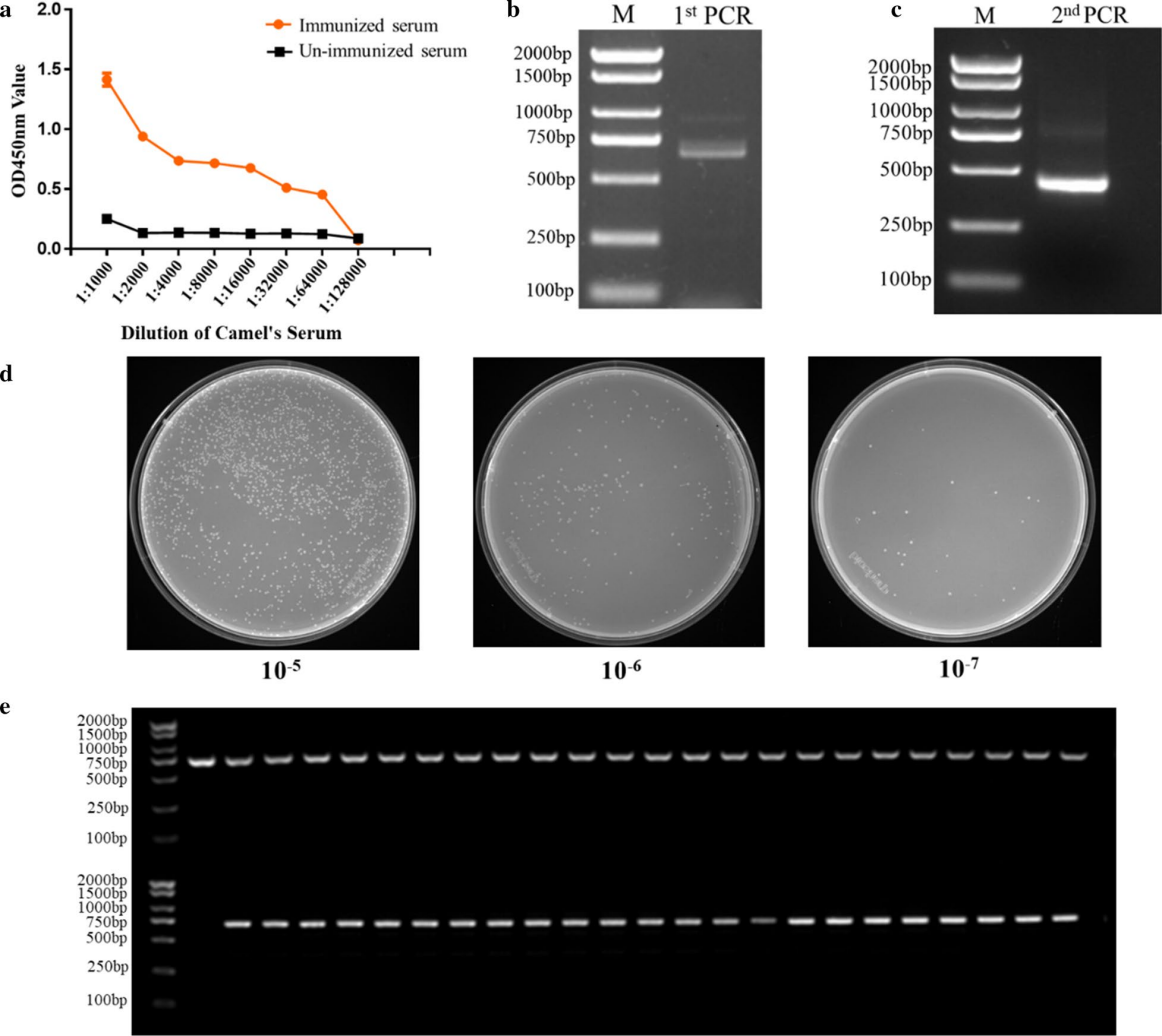
Nbs library construction against *S. Enteritidis*. **a** Titer of antibody against *S. Enteritidis* in the immunized camel serum. **b** The first round PCR with approximately 700 bp fragment. **c** The second round PCR with approximately 400 bp fragment. **d** Nbs library construction counted by tenfold gradient dilution. At the seventh dilution level, 23 single colonies present on the LB plate with 2% final concentration of glucose and 100 µg/mL ampicillin, indicating that 23 phages particles have invaded the TG1 cells, which finally show the phage display library was 2.3 × 10^9^ PFU/mL. **e** Identification of the correct insert rate of 48 colony by PCR with approximately 700 bp fragment

To ensure the safety of the immunized Bactrian camel, it was confirmed that there was no bacteria colony growing on the LB plate. After immunizing five times with *S. Enteriditidis*, the titer of antibody against *S. Enteriditidis* reached 1:64,000 (Fig. 1a), which indicated that the Bactrian camel was immunized with a robust immune response for the subsequent experimental needs. The total cellular RNA was isolated from around 200 mL of the peripheral blood and was reverse transcribed into cDNA and then was amplified to the fragments of 700 bp and 900 bp approximately in the first round PCR (Fig. 1b). Then, a target band of about 400 bp in size was amplified in the second PCR (Fig. 1c) and finally, the phage display library was constructed against *S. Enteriditidis* and its capacity reached about 2.3 × 10^9^ PFU/mL (Fig. 1d). The insertion of the VHH genes and the diversity of the library were confirmed where 48 individual clones were randomly selected for detection using colony PCR, suggesting an insertion rate of more than 98% (Fig. 1e). The sequencing result exhibited a great diversity (data not shown). These results indicated the successful development of a reliable phage display library against *S. Enteriditidis* screening nanobodies.

### Bio-screening and sequencing of Nbs against *S. Enteriditidis*

**Fig. 2:**
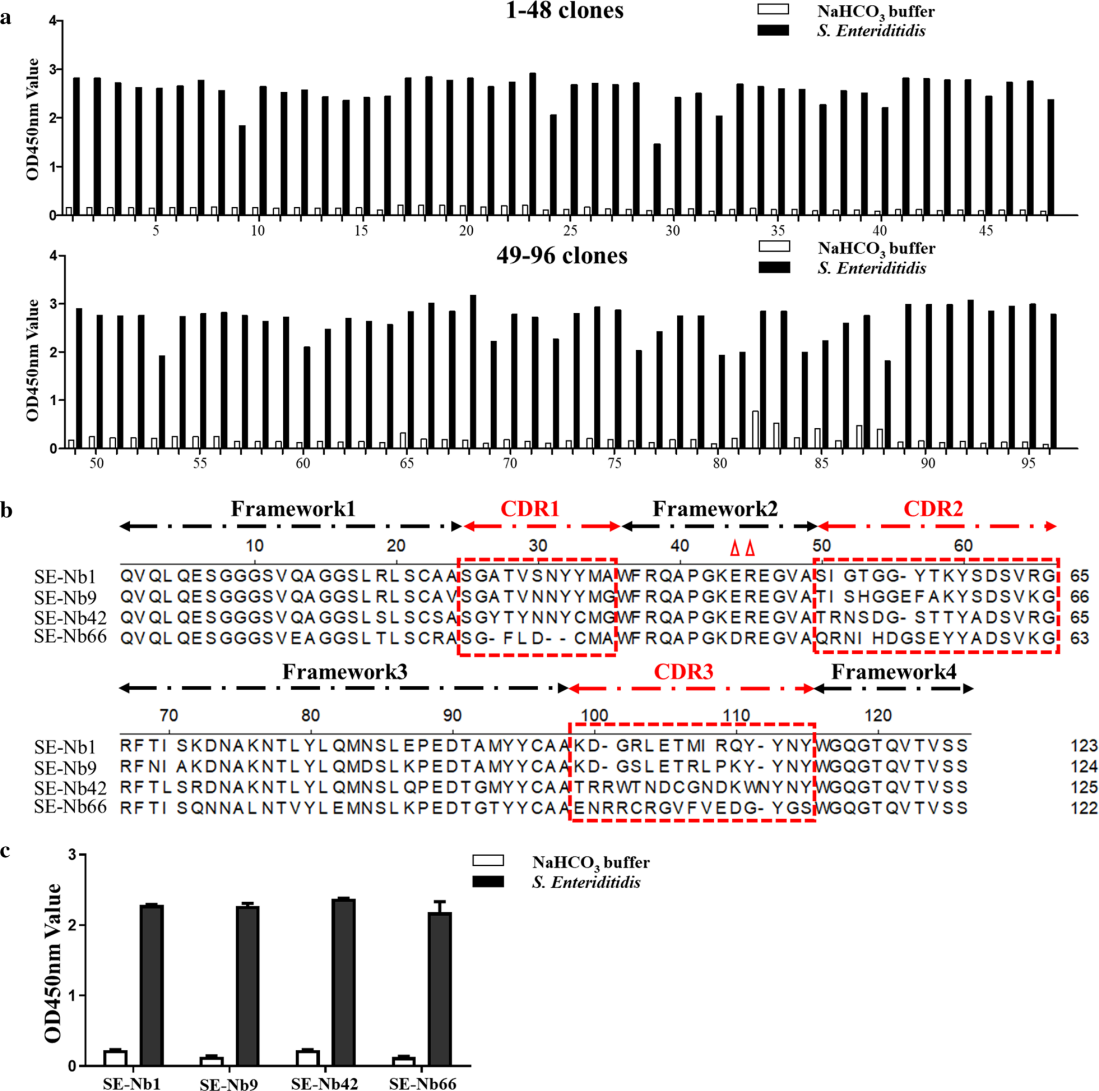
Screening nanobodies against *S. Enteritidis.* **a** Identification the periplasmic extract of 96 clones to specifically bound with the *S. Enteriditidis*, with positive rate of 100%. **b** Alignment and classification of the amino acid sequences of 4 screened nanobodies based on CDRs. **c** Determination the binding activity of 4 screened nanobodies with *S. Enteriditidis*

The four rounds of bio-panning indicated a continually elevated enrichment of phage particles was from the first round to the third round, but the enrichment was slightly lower than that was before the fourth round (Additional file 1: Figure S1). As a result, the binding of phage particles with *S. Enteriditidis* was highly enriched with the output increasing from 4.2 × 10^5^ PFU to 1.2 × 10^10^ PFU, and the ratio of the positive/negative clones (P/N) was found to increase from 54 to 1.2 × 10^4^ (Table 1). To analyze the binding capacity with *S. Enteriditidis*, 96 individual clones were randomly picked from the third round (48 clones) and the fourth round (48 clones). The results revealed that the periplasmic extract of 96 clones could specifically bind with *S. Enteriditidis* (Fig. 2a). Then, the positive clones were sequenced using the MP57 primer and subsequently classified based on the amino acid sequences of the CDRs. Finally, four different Nbs were selected and named SE-Nb1, SE-Nb9, SE-Nb42, and SE-Nb99 (Fig. 2b). It was noteworthy that the VHH sequence of SE-Nb1 was repeated 44 times in the 96 clones. Furthermore, all 4 nanobodies were found to show a high binding activity with *S. Enteriditidis* though iELISA (Fig. 2c).

**Table 1:** Enrichment of phage particles against *S. Enteritidis* specific nanobodies during four rounds of panning

Round of screening	Input (PFU/well)	P output (PFU/well)	N output (PFU/well)	Recovery(P/Input)	P/N
1st Round	5 × 10^11^	4.2 × 10^5^	8 × 10^3^	8.4 × 10^–7^	54
1nd Round	5 × 10^11^	3.6 × 10^7^	1.2 × 10^4^	7.2 × 10^–5^	3 × 10^3^
1rd Round	5 × 10^11^	7.2 × 10^9^	6 × 10^5^	1.44 × 10^–3^	1.2 × 10^4^
1th Round	5 × 10^11^	1.2 × 10^10^	5 × 10^6^	2.4 × 10^–2^	2.4 × 10^3^

### Expression, purification and characterization of the Nbs

**Fig. 3:**
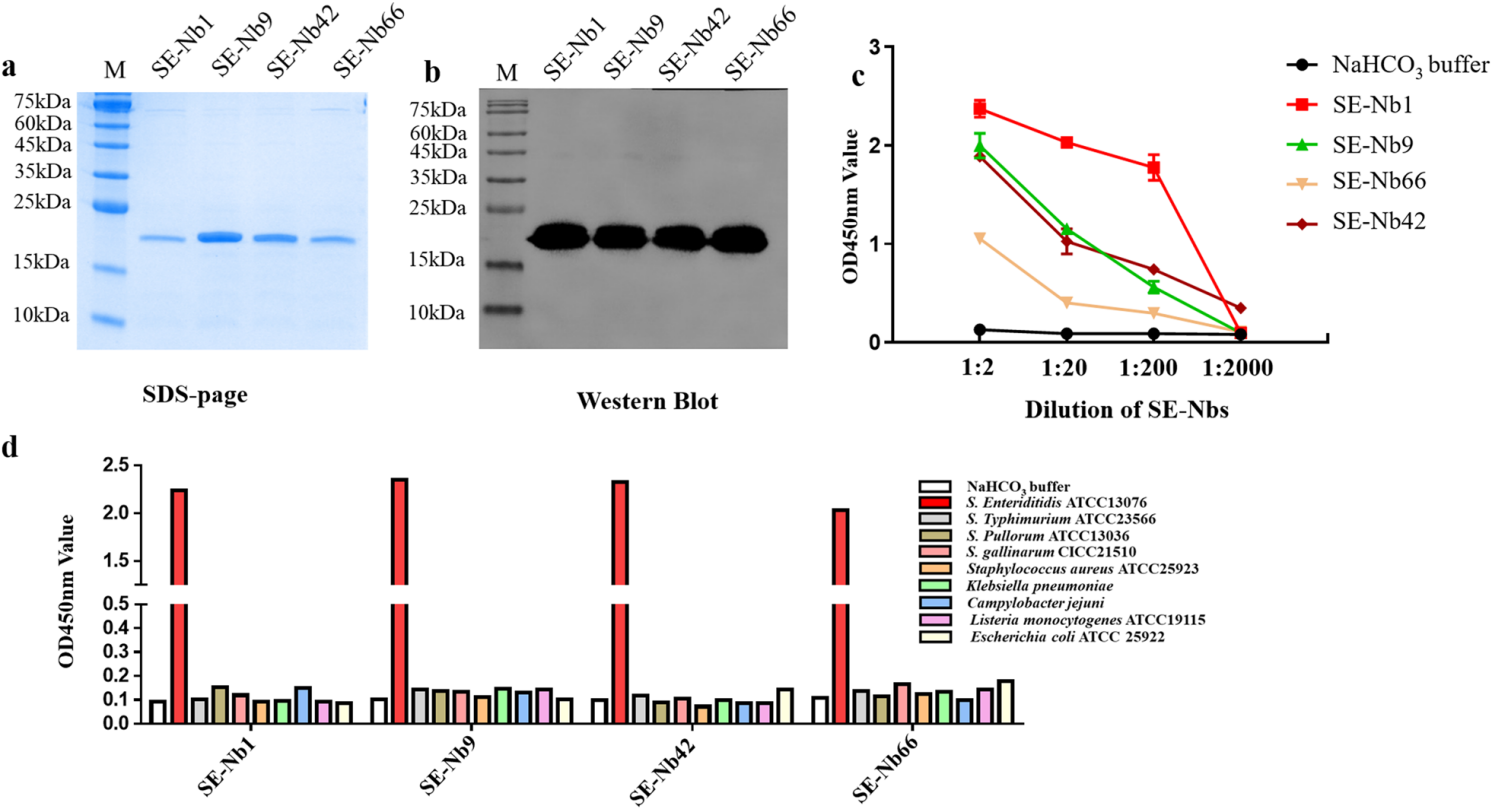
Expression, purification and identification of the 4 recombinant nanobodies against *S. Enteriditidis*. **a** Analysis of 4 recombinant nanobodies expression by SDS-page. **b** Determination of 4 recombinant nanobodies by Western blot. **c** Detection of the 4 recombinant nanobodies specifically binding to the *S. Enteriditidis* by the indirect ELISA. **d** Identification the 4 recombinant nanobodies to bind another strain of *S. Enteriditidis*, three *Salmonella* strains and five other non-*Salmonella* strains

Four VHH genes encoding SE-Nb1, SE-Nb9, SE-Nb42 and SE-Nb66 above were successfully ligated into the pET-25b vector. Four soluble proteins with the expected size of approximately 15 kDa were purified after induction with 0.2 mM IPTG for 16 h and determined by SDS-PAGE (Fig. 3a). Moreover, the western blot analysis showed that the four Nbs could react with the His-tag monoclonal antibody (Fig. 3b). The results of the iELISA revealed that SE-Nb1, SE-Nb9, SE-Nb42 and SE-Nb66 could maintain a high binding capacity with *S. Enteriditidis* (Fig. 3c). The cross-reactivity analysis indicated the Nbs could bind with *S. Enteriditidis*, but showed no reaction with the other three *Salmonella* strains or the five non-*Salmonella* strains (Fig. 3d, e).

### Production and characterization of the nanobody‑vHRP fusion against *S. Enteriditidis*

**Fig. 4:**
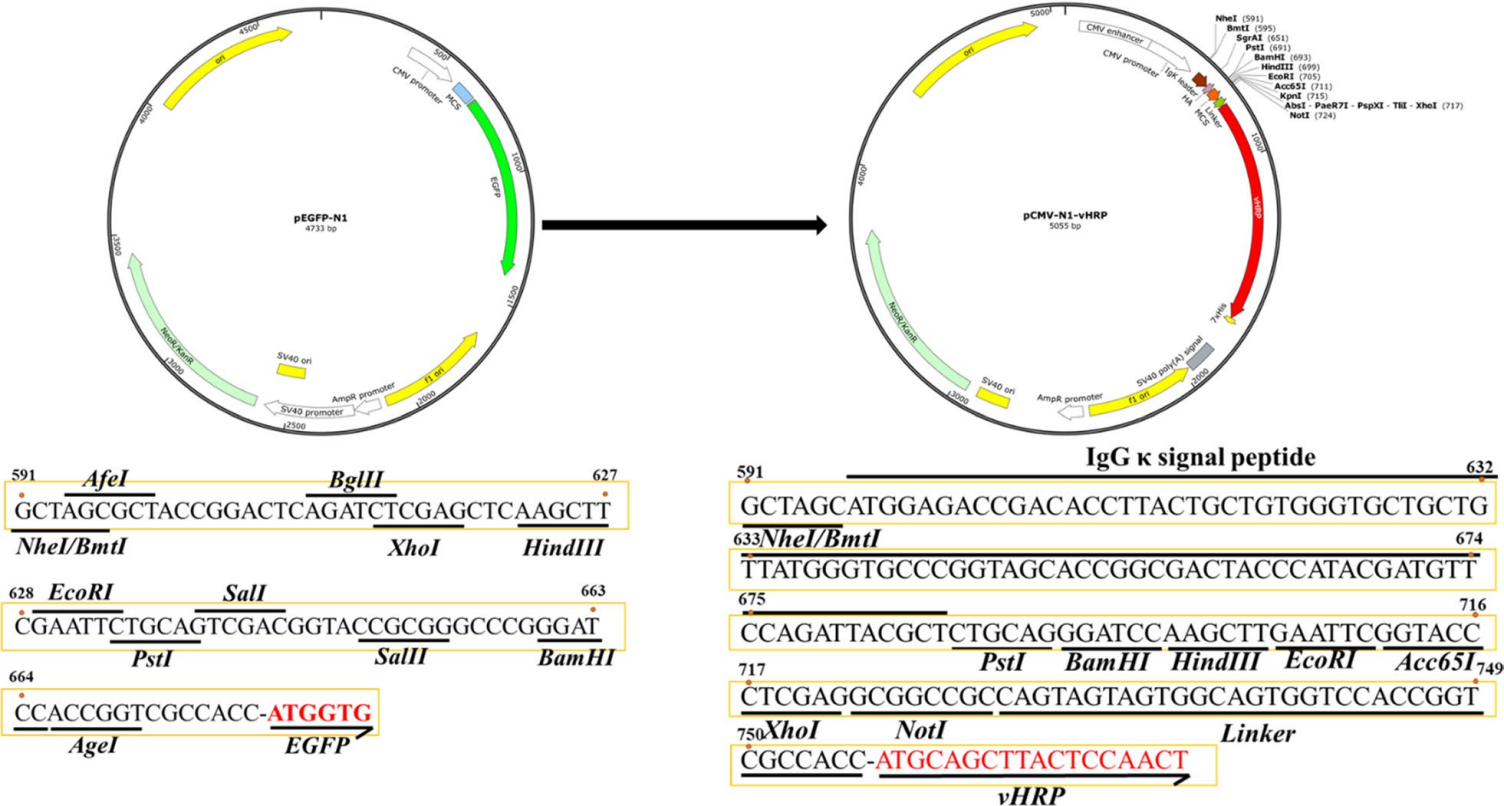
Construction of pCMV-N1-vHRP vector. The commercial vector pEGFP‑N1 changed into the vector to insert the main genes encoding IgG signal peptide, multiple cloning site, vHRP gene, HA and 7 × His flag

**Fig. 5:**
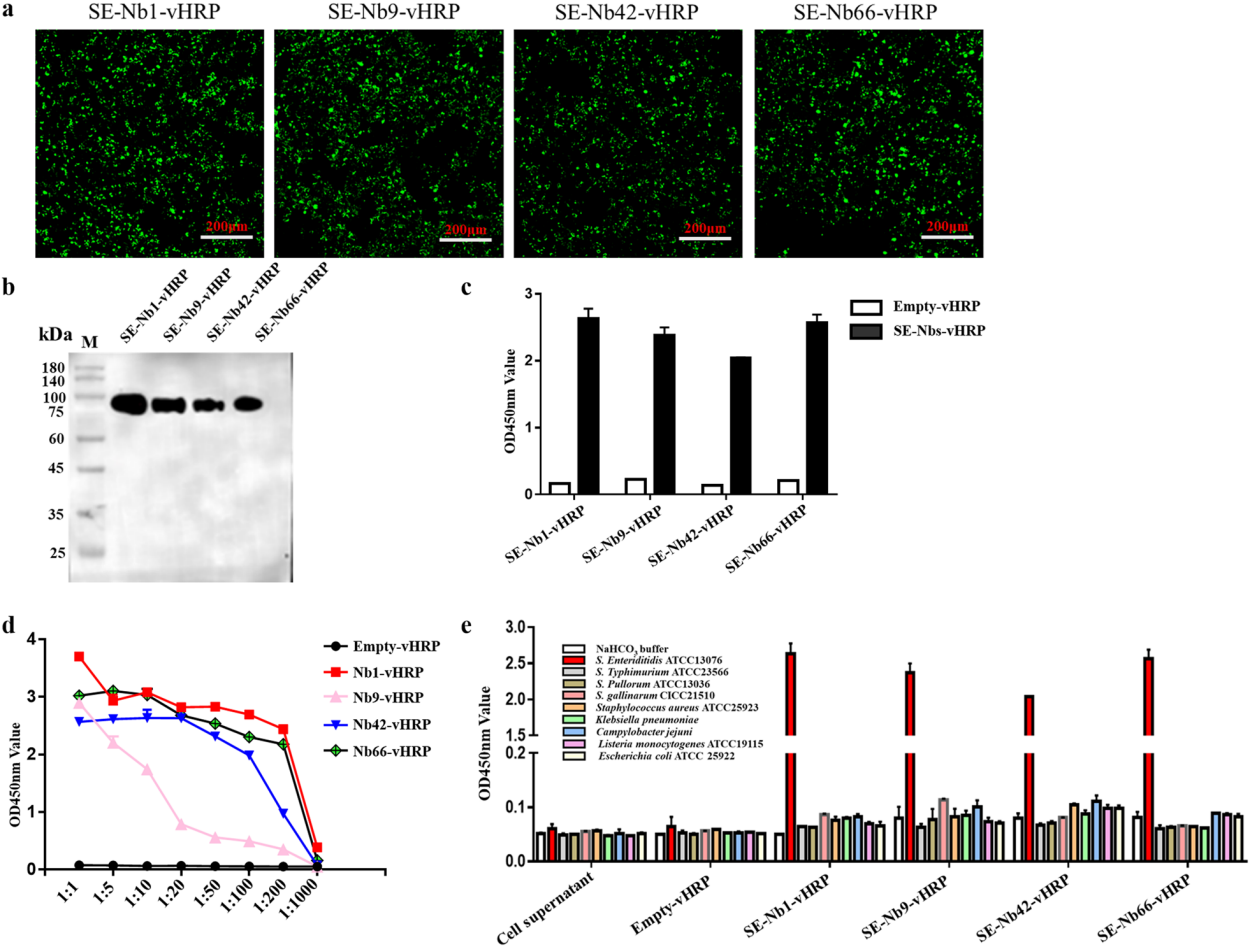
Production and characterization of the four nanobody‑vHRP fusions against *S. Enteriditidis.* **a** Detection of the four nanobody‑vHRP fusions expressed in the HEK293T cells by IFA. **b** Western blot analysis with anti-His monoclonal antibody. **c** The four nanobody‑vHRP fusions specifically binding with *S. Enteriditidis* by direct ELISA. **d** Determination the capacity of four nanobody‑vHRP fusions binding with the *S. Enteriditidis* by direct ELISA. **e** Identification the four nanobody‑vHRP fusions reaction with another strain of *S. Enteriditidis*, three *Salmonella* strains and five other *non-Salmonella* strains

The nanobody-vHRP fusions were produced by using as a backbone and then reconstructing it into a novel pCMV-N1-vHRP vector (Fig. 4a), capable of expressing the fusion protein of the signal peptide of human Ig kappa chain, HA tag, nanobodies coupled with codon-optimized HRP, and His tag in the HEK293T cells. The EGFP protein as a positive control was found to effectively express in the HEK293T cells by direct observation under a fluorescence microscope (Additional file 1: Figure S2). The four nanobody‑vHRP fusions were produced in the HEK293T cells using indirect immunofluorescence assay (Fig. 5a). The results of the western blot analysis and iELISA indicated that these nanobody‑vHRP fusions were secreted into the cell supernatant, named as SE-Nb1‑vHRP, SE-Nb9‑vHRP, SE-Nb42‑vHRP, SE-Nb66‑vHRP (Fig. 5b, c). The direct ELISA showed that the four nanobody‑vHRP fusions reacted with *S. Enteriditidis* (Fig. 5d). The cross-reactivity was characterized by a direct ELISA, with cell supernatant and supernatant of the transfected pCMV-N1-vHRP vector as a negative control. This indicated that the four nanobody‑vHRP was capable of binding with *S. Enteriditidis*, but not the other three *Salmonella* strains or the five *non-Salmonella* strains (Fig. 5e).

### Development of the double nanobody‑based sandwich ELISA to detect *S. Enteriditidis*

**Fig. 6:**
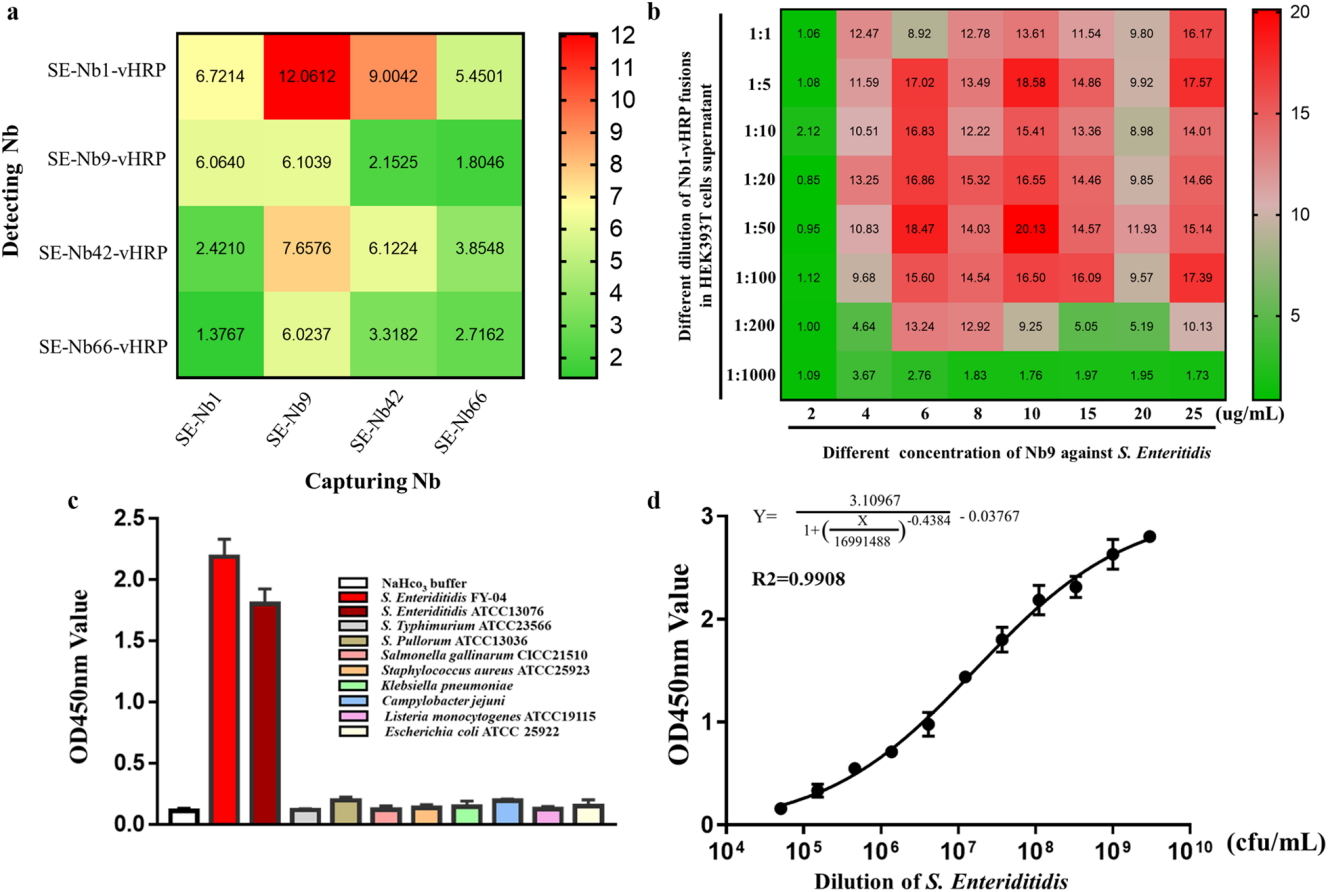
Development of the double nanobody‑based sandwich ELISA to detect *S. Enteriditidis*. **a** The Nb-pair comprising SE-Nb9 and SE-Nb1-vHRP were chosen as capturing and detecting antibodies, respectively. **b** The concentration of 10 μg/mL SE-Nb1 and SE-Nb1-vHRP (1:50) were optimized for detecting *S. Enteriditidis.* **c** The specificity and cross-reactivity analysis with two strains of *S. Enteriditidis*, three strains of other *Salmonella* and five other *non-Salmonella* strains. **d** The standard curve of the developed immunoassay to detect *S. Enteriditidis*

The double nanobody‑based sandwich ELISA was developed using SE-Nbs to capture antigen and SE-Nbs‑vHRP to detect the captured antigen. The result of the orthogonal assay was presented in Additional file 1: Table S2, which indicated that in the sandwich ELISA SE-Nb9 and SE-Nb1‑vHRP as the capture and detecting antibody, respectively were an optimal pair with the highest P/N value reach to 12.06 (Fig. 6a). The checkerboard titration showed that the best P/N value could reach 20.13, when 1 μg/well SE-Nb9 and SE-Nb1‑vHRP fusion at a dilution of 1:50 could be used to establish the double nanobody-based sandwich ELISA for monitoring *S. Enteriditidis* (Fig. 6b) and the absorbance value at 450 nm was shown in Additional file 1: Table S3. The specificity and cross-reactivity analysis implied that the sandwich ELISA showed excellent reactivity with *S. Enteriditidis 13076* and *S. Enteriditidis* FY-04 strains, whereas there was no significant response for the other three *Salmonella* strains and five *non-Salmonella* strains (Fig. 6c). A four parameter logarithmic equation for a non-linear curve was fitted between the absorbance value of 450 nm and the count of *S. Enteriditidis.* The regression equation was determined as:

$$\mathrm{Y}=\frac{3.10967}{1+(\frac{X}{16991488}{)}^{-0.4384}}-0.03767$$,

(R^2^ = 0.9908), with the Y-axis and X-axis represented the absorbance value at 450 nm and the concentration of bacteria (Fig. 6d). The absorbance values of the blank wells plus 3 times the standard deviation was treated as the cut-off value (OD450 nm value = 0.188) and the principle for evaluating the LOD of the established sandwich ELISA. Lastly, the LOD was calculated though the regression equation above and found to be 5 × 10^4^ CFU/mL. It indicated that the value of OD450 nm > 0.188 represented the sample containing *S. Enteriditidis* and the number of *S. Enteriditidis* over 5 × 10^4^ CFU/mL. As a result, the developed sandwich ELISA was found to demonstrate good reproducibility and reliability (data not shown).

### Detection of *S. Enteriditidis* spiked in milk

To verify the credibility of the established sandwich ELISA, the procedures monitoring the various concentrations of *S. Enteriditidis* spiked in the dairy product of skimmed milk were designed (1 × 10^6^, 1 × 10^7^ and 1 × 10^8^ CFU/mL). The recovery assay revealed the recovery value to range from 97.02% to 108.59%, indicating that the developed sandwich ELISA was feasible for detecting *S. Enteriditidis* in the milk samples as shown in Table 2. The sensitivity of the established sandwich ELISA was tested in the spiked milk after an enrichment step. After 8 h of enrichment, 10 CFU/mL of *S. Enteriditidis* could be detected in the milk using the developed sandwich ELISA with no reactivity in the control well as evident in Table 3. As a result, this is a practical and promising immune detection tool applicable for monitoring *S. Enteriditidis* in skimmed milk with good sensitivity and specificity.

**Table 2:** Recovery of *S. Enteriditidis* in milk sample by the developed assay

Sample	Spiked (CFU/mL)	Detection (CFU/mL)	Recovery^a^ (%)	CV^b^ (%)
Skimmed milk	1 × 10^6^	1.08 × 10^6^ ± 1.12 × 10^5^	108.59%	10.32%
	1 × 10^7^	9.7 × 10^6^ ± 8.25 × 10^5^	97.02%	8.51%
	1 × 10^8^	1.01 × 10^7^ ± 8.38 × 10^5^	101.06%	8.3%

^a^Each assay was repeated three times, the result of the recovery was the average of three replicates

^b^CV was the ratio of the standard deviation to the mean

**Table 3:** Detection of *S. Enteriditidis* in skimmed milk after different enrichment period

Sample	Control^a^	Enrichment period^b^ (CFU/mL)				
Skimmed milk		6 h	8 h	10 h	12 h	14 h
	ND^**c**^	ND	1.91 × 10^5^ ± 2.57 × 10^4^	5.07 × 10^6^ ± 4.92 × 10^5^	9.6 × 10^6^ ± 8.73 × 10^5^	2.75 × 10^7^ ± 4.15 × 10^6^

^a^‘Control’ is skimmed milk sample without *S. Enteriditidis* used as blank

^b^Each assay was repeated three times, the result shows the average of three replicates

^c^ND, Not Detectable

### Detection of *S. Enteriditidis* colonized in vivo in the Chicken

**Fig. 7:**
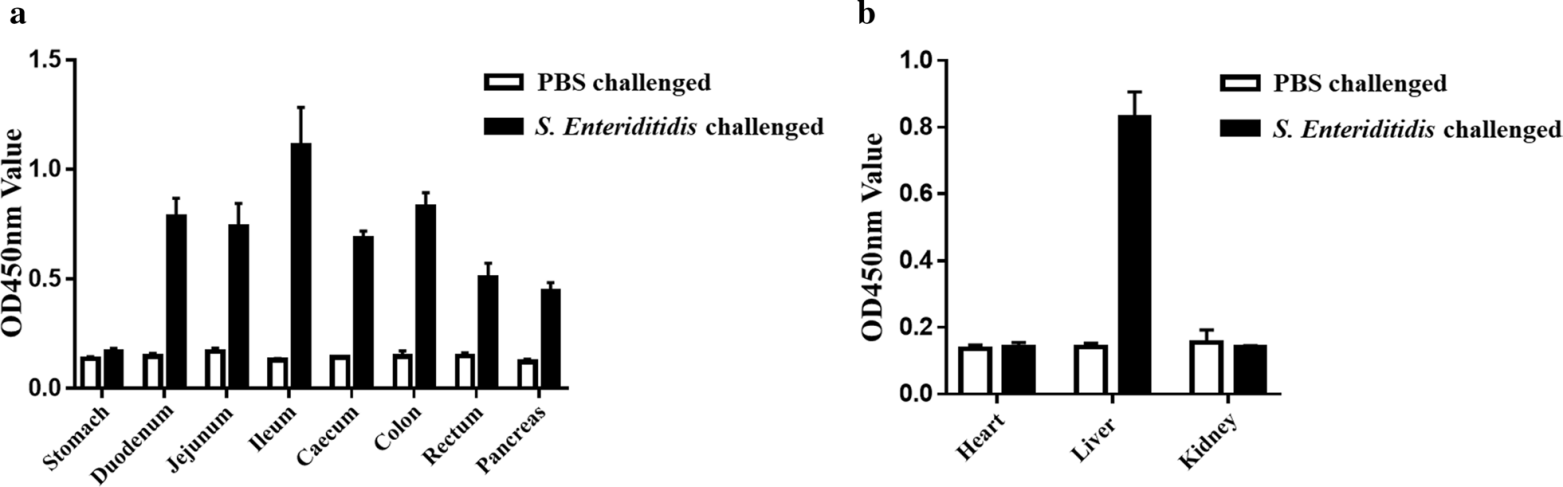
Detection of *S. Enteriditidis* of colonized in vivo in challenged Chickens**. a** Analysis the colonization of *S. Enteriditidis* in gastrointestinal tract. **b** Detection of *S. Enteriditidis* in other organs

The colonization of *S. Enteriditidis* was investigated in the intestinal tract and organs of the neonatal chicken with an oral challenge using the established sandwich ELISA. After 8 h of enrichment, all the selected organs and intestinal tract in the chickens were assessed by the developed ELISA assay, indicating that the *S. Enteriditidis* was maintained in almost all parts of the intestinal tract, including the duodenum, jejunum, ileum, caecum, colon, rectum and pancreas, but not in the stomach (Fig. 7a). In addition, the colonization of *S. Enteriditidis* in the other organs were also detected using the same method. The liver was particularly found to colonize with some *S. Enteriditidis* presenting a high value at 450 nm, which suggested that the liver was an organ with a risk of colonization and transmission of *S. Enteriditidis*. Moreover, there was no *S. Enteriditidis* in heart and kidney (Fig. 7b). The results presented though the ELISA assay were approximately consistent with the results of the real-time PCR. Furthermore, plate counting method indicated the isolation of the *S. Enteriditidis* in the positive sample as shown in Table 4.

**Table 4:** Comparisons of the developed sandwich ELISA with real-time PCR and plate culture method by detecting the *S. Enteriditidis* colonization in challenged chicken

Different assay for detecting *S. Enteriditidis*	Gastrointestinal tract								Other organs		
	Stomach	Duodenum	Jejunum	Ileum	Caecum	Colon	Rectum	Pancreas	Heart	Liver	Kidney
Developed sandwich ELISA	**−**	** + **	** + **	** + **	** + **	** + **	** + **	** + **	**−**	** + **	**−**
Real-time PCR	** + **	** + **	** + **	** + **	** + **	** + **	** + **	** + **	**−**	** + **	**−**
Plate isolation	**−**	** + **	** + **	** + **	** + **	** + **	** + **	** + **	**−**	** + **	**−**

Note “**−**” represents negative for not detectable, “ + ” represents positive for detectable

## Discussion

*S. Enteritidis* as a major zoonotic pathogen cause due to the transmission of bacteria through the ingestion of contaminated food including dairy products, meats, eggs, seafood, vegetables and many other foods, which lead to human gastroenteritis [31–33]. To date, the conventional culture-based methods which are referred to as the “gold standard” to detect *S. Enteritidis* are labor-intensive, time-consuming, low throughput and lack efficiency [30]. Although the nucleic acid-based techniques are used, they require special equipment, high cost, trained operator personnel, the presence of aerosol pollution for “false” positive [32], such as polymerase chain reaction [34], real-time PCR [35], nucleic acid sequence-based amplification [36], loop-mediated isothermal amplification [30], recombinase polymerase amplification [37], recombinase aided amplification [38], rolling circle amplification [39], and its extension to the novel method combined with the CRISPR-Cas system [40, 41]. Furthermore, the nucleic acid-based approaches assessing specific serovars and subtypes necessitate the use of specially designed probes as well as the additional processes.

The polyclonal and monoclonal antibodies based on immunoassays are determined by the capacity of the particular antibody to bind to its specific antigen. Nonetheless, the preparation of antibodies is troublesome, with poor stability between the batches and the half-life and tolerance worried in extreme conditions [42]. Hence, a novel antibody in the immunoassays would be challenged to overcome the drawback of the conventional antibodies for detecting pathogenic bacteria.

Nanobodies are endowed with numerous excellent characteristics like high affinity, thermal stability, easy modification and production. This study developed an improved nanobody-horseradish peroxidase-based sandwich ELISA to detect *S. Enteritidis* rapidly with sensitivity. The capture antibody of SE-Nb9 was easily expressed in the high-yield *E. coli* system with purification of convenient compared to conventional antibodies. The SE-Nb1-vHRP as detection antibody can directly bind *S. Enteritidis* captured by SE-Nb9, reducing the use of commercial secondary antibodies and shortening the detection time in the sandwich ELISA. Moreover, the HEK293T cell lines which stably express the SE-Nb1-vHRP can be modulated into a stable cell line by cell domestication technology, with reducing the cost and simplifying the production.

Compared with the other immunoassays reported, the developed sandwich assay was found to show much superiority in terms of sensitivity [16, 43, 44]. Such as He.et al. [16] utilized the commercialized *Salmonella*-specific polyclonal antibody as the capture and nanobody-13 as detection antibody to establish the sandwich immunoassay for detecting *S. Enteritidis* in milk sample, in which the LOD reach to 1.4 × 10^5^ CFU/mL *S. Enteritidis* and require the recognition of HA tag on nanobody with HRP conjugated anti-HA antibody for color reaction. Herein, the double nanobodies based immunoassay with the LOD of 5 × 10^4^ CFU/mL is more sensitive than above methods and it does not require the commercial horseradish peroxidase labeled secondary antibody. The fact that this assay was utilized to detect *S. Enteritidis* is its biggest strength, which implied that both time and cost could be saved, as well as the shelf life could be prolonged with and the ability to tolerate extended working periods in harsh environments [42]. Using the established assay, the colonization of *S. Enteriditidis* was explored in infected chicken. We found that *S. Enteriditidis* could survive practically in all the sections of the intestinal tract and the liver with a variety of strains. Moreover, the results were found to be realistic, reproducible, and aligned well with that of the real-time PCR. The plate culture approach may be used to successfully isolate *S. Enteriditidis* from positive samples of the challenged chicken, with a 100% match to result of the sandwich ELISA*.* The sandwich assay speculated to be a supplementary technology for effective isolating *S. Enteriditidis* from the poultry clinical samples. Of course, larger numbers and more complex clinical samples are needed to verify this method. These features make this strategy suitable for the on-site detection for risk assessment of *S. Enteriditidis* associated with them. At last, the developed sandwich ELISA based on the double nanobodies believed to have great potential for monitoring *S. Enteriditidis* in milk and poultry products, and can extend its application.

## Conclusion

In summary, this study screened four specific nanobodies against *S. Enteriditidis* using the phage display technology. The SE-Nb1 and SE-Nb9-vHRP are employed as the capture and detection reagents respectively for developing a sandwich ELISA to detect *S. Enteriditidis.* This newly developed sandwich ELISA offers a simple alternative method with low-cost production, high throughput, and rapid detection. This method can be used to detect *S. Enteriditidis* in milk as low as 5 × 10^4^ CFU/mL and approximately 10 CFU/mL of *S. Enteritidis* can be detected after 8 h of enrichment step. In addition, the developed sandwich can analyze the colonization of *S. Enteriditidis* in the infected chicken for risk assessment. Therefore, the developed sandwich ELISA is applicable for determining *S. Enteritidis* in spiked milk and has immense prospects in the poultry industry for controlling food safety.

## Supplementary Information

**Additional file 1.** Additional figures and tables.
