# Supplementary material for: Development of nanobody-horseradish peroxidase-based sandwich ELISA to detect Salmonella Enteritidis in milk and in vivo colonization in chicken
Source: J Nanobiotechnology. 2022 Mar 31;20:167. doi: 10.1186/s12951-022-01376-y (PMC8973953; doi:10.1186/s12951-022-01376-y)
Supplement: Supplementary file 1 — Additional file 1. Additional figures and tables. [file 12951_2022_1376_MOESM1_ESM.docx]

**Supplementary Information**

**Development of nanobody-horseradish peroxidase-based sandwich ELISA to detect *S. Enteritidis* in milk and in vivo colonization in chicken**

Kui Gu^1,2^, Zengxu Song^1,2^, Changyu Zhou^1,2^, Peng Ma^1,2^, Chao Li^1,2^, Qizhong Lu^3^, Ziwei Liao^1,2^, Zheren Huang^1,2^, Yizhi Tang^1,2^, Hao Li^1,2^, Yu Zhao^1,2^, Wenjun Yan^1,2^, Changwei Lei^1,2^ and Hongning Wang^1,2^

**Table S1** Primers used for this work. The underlying sequences in the primers were restriction sites.

| Primers | Sequences (5’-3’) |
| --- | --- |
| CALL001 | GTCCTGGCTGCTCTTCTACAAGG |
| CALL002 | GGTACGTGCTGTTGAACTGTTCC |
| VHH-FOR ( *PstI* ) | CAGGTGCAGCTGCAGGAGTCTGGGGGAGR |
| VHH-REV ( *NotI* ) | CTAGTGCGGCCGCTGAGGAGACGGTGACCTGGGT |
| MP57 | TTATGCTTCCGGCTCGTATG |
| GIII | CCACAGACAGCCCTCATAG |
| SE-Nb-F ( *BamHI* ) | TATGGATCCGCAGGTGCAGCTGCAGGAG |
| SE-Nb-R ( *HindIII* ) | AGTAAGCTTTGAGGAGACGGTGACCTG |
| EGFP-F ( *PstI* ) | ATACTGCAGATGGTGAGCAAGGGCG |
| EGFP-R (*HindIII* ) | CCCAAGCTTCTTGTACAGCTCGTCCATG |
| Nb‑vHRP-F ( *PstI* ) | ATACTGCAGCAGGTGCAGCTGCAGGAG |
| Nb‑vHRP-R (*HindIII* ) | AGTAAGCTTTGAGGAGACGGTGACCTG |
| qPCR-F | CGGTCGTAGTGGTGTCT |
| qPCR-R | GCGGCAGTTCTTCGTAA |

**Table S2** Determination of the best pairs of nanobodies, as the capture antibody, and nanobody-HRP fusions as the detection antibody for the sandwich ELISA to detect *S. Enteriditidis* by orthogonal assay

|  | OD450 | SE-Nb1 | SE-Nb9 | SE-Nb42 | SE-Nb66 |
| --- | --- | --- | --- | --- | --- |
| SE-Nb1-vHRP | Positive | 0.9094 | 1.6265 | 0.9617 | 0.5273 |
|  | negative | 0.1353 | 0.1349 | 0.1068 | 0.0968 |
|  | P/N | 6.7214 | **12.0612** | 9.0042 | 5.4501 |
| SE-Nb9-vHRP | Positive | 0.6155 | 0.5964 | 0.1877 | 0.1524 |
|  | negative | 0.1015 | 0.0977 | 0.0872 | 0.0845 |
|  | P/N | 6.0640 | 6.1039 | 2.1525 | 1.8046 |
| SE-Nb42-vHRP | Positive | 0.3287 | 0.9381 | 0.7304 | 0.4792 |
|  | negative | 0.1358 | 0.1225 | 0.1193 | 0.1243 |
|  | P/N | 2.4210 | 7.6576 | 6.1224 | 3.8548 |
| SE-Nb66-vHRP | Positive | 0.1402 | 0.5334 | 0.2638 | 0.2251 |
|  | P/N | 0.1358 | 0.0886 | 0.0795 | 0.0939 |
|  | Positive | 1.3767 | 6.0237 | 3.3182 | 2.7162 |

**Table S3** Optimized amount of SE-Nb9 as the capture antibody and dilution of SE-Nb1-vHRP fusions in the medium as the detection antibody using the developed sandwich ELISA.

|  | **OD450** | **25ug/mL** | **20ug/mL** | **15ug/mL** | **10ug/mL** | **8ug/mL** | **6ug/mL** | **4ug/mL** | **2ug/mL** |
| --- | --- | --- | --- | --- | --- | --- | --- | --- | --- |
| **Nb1-HRP**  **initial concentration** | **Positive** | 2.23 | 1.67 | 1.61. | 1.94 | 1.70 | 1.40 | 1.42 | 0.14 |
|  | **negative** | 0.13 | 0.17 | 0.14 | 0.13 | 0.13 | 0.16 | 0.11 | 0.13 |
|  | **P/N** | 16.17 | 9.80 | 11.54 | 13.61 | 12.78 | 8.92 | 12.47 | 1.06 |
| **Nb1-vHRP**  **（1：5）** | **Positive** | 2.03 | 1.60 | 1.80 | 1.97 | 2.09 | 2.16 | 1.71 | 0.14 |
|  | **negative** | 0.12 | 0.16 | 0.12 | 0.11 | 0.15 | 0.13 | 0.15 | 0.13 |
|  | **P/N** | 17.57 | 9.92 | 14.86 | 18.58 | 13.49 | 17.02 | 11.59 | 1.08 |
| **Nb1-vHRP**  **（1：10）** | **Positive** | 2.11 | 1.58 | 1.51 | 2.22 | 1.80 | 2.39 | 1.39 | 0.30 |
|  | **negative** | 0.15 | 0.18 | 0.11 | 0.14 | 0.15 | 0.14 | 0.13 | 0.14 |
|  | **P/N** | 14.00 | 8.98 | 13.36 | 15.41 | 12.22 | 16.83 | 10.51 | 2.12 |
| **Nb1-vHRP**  **（1：20）** | **Positive** | 2.02 | 1.54 | 1.89 | 2.20 | 2.08 | 2.29 | 1.48 | 0.12 |
|  | **negative** | 0.14 | 0.16 | 0.13 | 0.13 | 0.14 | 0.14 | 0.11 | 0.14 |
|  | **P/N** | 14.66 | 9.85 | 14.46 | 16.55 | 15.32 | 16.86 | 13.25 | 0.85 |
| **Nb1-vHRP**  **（1：50）** | **Positive** | 2.12 | 1.34 | 1.79 | 2.45 | 2.05 | 2.27 | 1.38 | 0.11 |
|  | **negative** | 0.14 | 0.11 | 0.12 | 0.12 | 0.15 | 0.12 | 0.13 | 0.11 |
|  | **P/N** | 15.14 | 11.93 | 14.57 | **20.13** | 14.03 | 18.47 | 10.83 | 0.95 |
| **Nb1-vHRP（1：100）** | **Positive** | 2.13 | 0.99 | 1.84 | 2.40 | 2.07 | 2.27 | 1.37 | 0.12 |
|  | **negative** | 0.12 | 0.10 | 0.11 | 0.15 | 0.14 | 0.15 | 0.14 | 0.11 |
|  | **P/N** | 17.39 | 9.57 | 16.09 | 16.50 | 14.54 | 15.60 | 9.68 | 1.12 |
| **Nb1-vHRP**  **（1：200）** | **Positive** | 1.38 | 0.88 | 0.82 | 1.28 | 1.79 | 2.03 | 0.64 | 0.14 |
|  | **negative** | 0.14 | 0.17 | 0.16 | 0.14 | 0.14 | 0.15 | 0.14 | 0.14 |
|  | **P/N** | 10.13 | 5.19 | 5.05 | 9.25 | 12.92 | 13.64 | 4.64 | 1.0 |
| **Nb1-vHRP**  **（1：1000）** | **Positive** | 0.21 | 0.26 | 0.28 | 0.28 | 0.25 | 0.40 | 0.48 | 0.15 |
|  | **negative** | 0.12 | 0.13 | 0.14 | 0.16 | 0.14 | 0.15 | 0.13 | 0.13 |
|  | **P/N** | 1.73 | 1.95 | 1.97 | 1.76 | 1.83 | 2.76 | 3.67 | 1.09 |


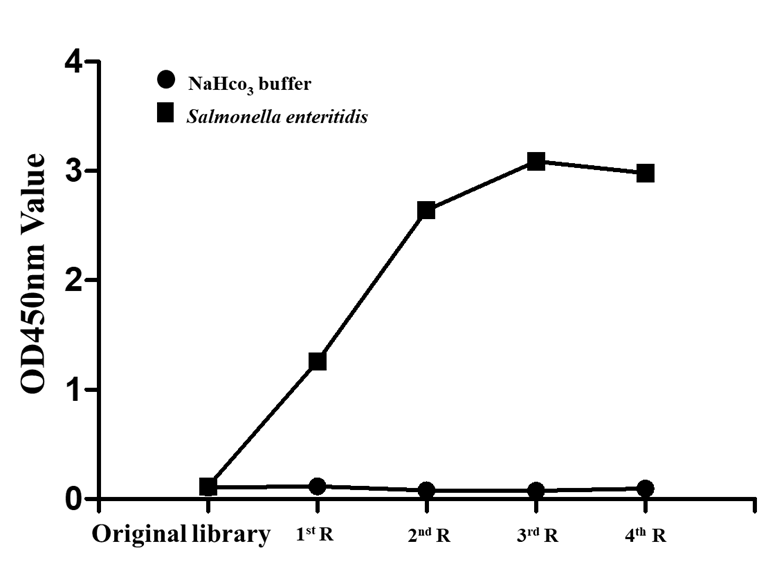


**Figure S1** Detection of the enrichment of phage particles against *S. Enteriditidis* by phage ELISA


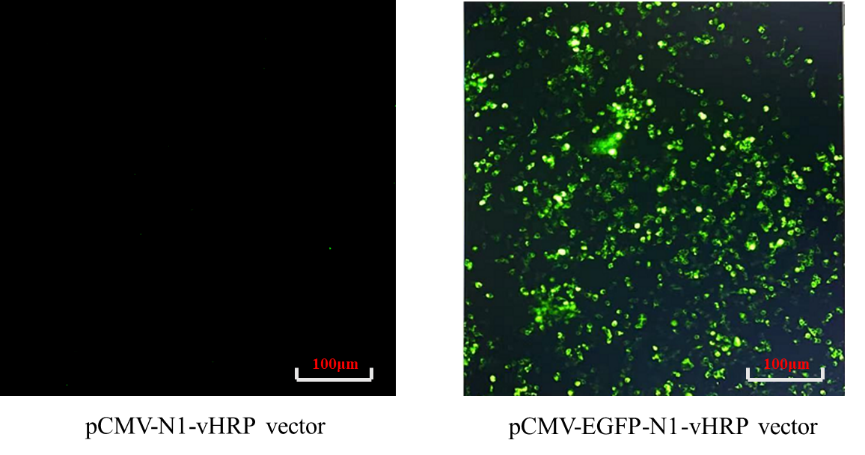


**Figure S2** The EGFP-vHRP fusion protein of identification in HEK-293T cell by direct observation via fluorescence microscopy.
